# The effect of nigella *sativa* oil on the prevention of phlebitis induced by chemotherapy: a clinical trial

**DOI:** 10.1051/bmdcn/2019090320

**Published:** 2019-08-27

**Authors:** Niaz Behnamfar, Zohreh Parsa Yekta, Faraz Mojab, Seyyed Mohammad Kazem Naeini

**Affiliations:** 1 Department of Nursing, Faculty of Nursing and Midwifery, Tehran Medical Sciences, Islamic Azad University Tehran Iran; 2 Department of Nursing management, Faculty of Nursing and Midwifery, Tehran Medical Sciences, Islamic Azad University Tehran Iran; 3 Department of Pharmacognosy, School of Pharmacy, Shahid Beheshti University of Medical Sciences Tehran Iran; 4 Department of Biostatistics, Tehran Medical Sciences, Islamic Azad University Tehran Iran

**Keywords:** Nigella *Sativa* oil, Prevention, Phlebitis, Chemotherapy

## Abstract

Introduction: Phlebitis, that disrupts chemotherapy, is the inflammation of the vein and the most common complication of intravenous injection of chemotherapy drugs.

Aim: the aim was determine the effect of topical application of N. *sativa* oil on the prevention of phlebitis caused by chemotherapy.

Methods: This single-blind clinical trial was conducted on 60 cancer patients. In the intervention group, five drops of N. *sativa* oil was applied on the distal area of the catheter, two times per day and every 12 hours from the first day of chemotherapy to the third day; no intervention was conducted for the subjects in the control group.

Results: Results showed that there was a significant difference between the two groups at 60 and 72 hours in regard with incidence of phlebitis. There was a statistically significant difference between the two groups at 12 and 72 hours in terms of severity (degree) of phlebitis.

Conclusion: topical application of N. *sativa* oil is effective in the prevention of chemotherapy-induced phlebitis.

## 1. Introduction

Intravenous chemotherapy is the most effective treatment for cancer in clinical settings [1, 2]. Intravenous catheters are used in cancer patients for hydration, administration of medications, administration of nutritional solutions, blood transfusion and blood products. Although the application of venous catheters has many benefits to patients in the oncology sections, they may cause various complications such as phlebitis, drug leakage, thrombophlebitis, air embolism, circulatory overload, hemorrhage, and infection [3–5]. Meanwhile, phlebitis is the most common complication [6–8]. Previous studies have reported the incidence of chemotherapy-induced phlebitis to be 35-65% in patients with cancer [1]. Phlebitis means inflammation of the vein [9, 10] and inflammatory response to intravenous infusion, leading to various types of tissue damage such as pain, warmth, redness, swelling, rigidity of the injection site, and ultimately fever [11]. The majority of chemotherapy drugs have the potential to cause phlebitis. Phlebitis caused by chemotherapy is a common complication in cancer patients; it causes several health problems for patients and affects the normal process of chemotherapy [1]. Phlebitis induced by intravenous anticancer agents might be caused by PH, Osmotic pressure of chemotherapy solutions, size of the used vein, size and type of the intravenous catheter and infusion periods [12, 13]. Therefore, while using of intravenous catheters for intravenous injection of chemotherapy drugs, attention should be paid to some of the aspects of nursing infusion standards, especially when used in vesicant or hyper-osmolar drugs, or when the duration of infusion is prolonged up to more than 60 minutes [14]. Phlebitis of peripheral vein catheters is an important factor of mortality in oncology patients [15]. Peripheral vein catheters which are normally applied in the oncology wards, can develop infections in the area of phlebitis to cellulitis and ultimately severe sepsis [15]. Therefore, it is essential for the nursing team to recognize these risk factors and apply care based on scientific evidence and executive protocols [16]. Phlebitis can be considered as a temporary or permanent limiting factor of treatment continuation in the cancer patients, after its occurrence peripheral vein catheter should be removed immediately. Depending on the intensity of the inflammatory process in the venipuncture area, vascular endothelium suffers from irreversible damage, including phlebo- sclerosis, and that portion of the vein can no longer be used to administer new intravenous line or even blood sampling. Therefore, prevention of phlebitis and the control of its inflammatory symptoms is essential, especially in patients whose venous network is highly susceptible to chemotherapy [17]. Phlebitis treatment involves removal of intravenous catheters and re-establishing in another site, applying warm and wet compresses in the damaged area and use of sterile techniques during catheterization [18]. Herbal therapy is a popular method in the last decades [19, 20] Various methods have been proposed to prevent phlebitis induced by chemotherapy, including topical application of sesame oil [21], aloevera [22], steroid ointment [23], topical application of chamomile [17] and heparin lack [24]. Medicinal plants are currently used as complementary and alternative therapies with minimal side effects, among which Nigella Sativa (N. *sativa*) has a rich medical history [25]. Known with scientific name of Nigella Sativa L. and belonging to the family of Ranunculacea. this plant is among the various medicinal species known as an amazing and miraculous plant with a wide range of medicinal activities [26]. Qver the last thousands of years, N. *sativa* has been used to treat various diseases such as asthma, hypertension, diabetes, inflammation, cough, headache, eczema, fever and dizziness. Various studies have shown its anti-inflammatory, anti-bacterial, antihista- minic, anti-diabetic, and anti-cancer properties [27].

N. *sativa* widely use evidence showed its anti-inflammatory effect is comparable effect of of 100 mg/kg aspirin by its inhibitory effects on Carrageenan induced paw edema [28]. Also, evidences show N. *sativa* anti-inflammatory therapeutic effects on psoriasis (by produced the ethanolic extract) [29], Acne vulgaris (as an oil lotion 10% significantly reduced mean lesion count of and pustules after 2 months of therapy) [30, 31], Vitiligo (by Lyophilized seed extract of N. *sativa* and its active ingredient) [32, 33], Hypersensitivity reactions (by using carbonyl fraction of N. *sativa* and its active components, thymoquinone and nigellone) [34, 35] and Skin cancers (by enhancement of the natural killer (NK) cell in anti-cancer program) [36, 37].

Active compounds have been extracted from the seed and oil of N. *sativa*, including thymoquinone, thymohydroquinone, di- thymoquinone, thymol, carvacrol, Nigellimine-N-Oxide, nigel- licine, nigellidine and alpha hederine [38]. The most beneficial therapeutic properties of this plant is due to the presence of phenolic compounds, especially thymoquinone [39]. The mechanism of anti-inflammatory action of N. *sativa* oil and thymiquinone is realized through inhibiting cyclo-oxygenase and 5-lipoxygenase pathways and preventing the production of thromboxane B2 and leukotriene B4 [40–42]. A study by Hadi et al in 2016 showed that N. *sativa* oil could be helpful in reducing the inflammation process and it can be used as a supplementary treatment in patients with rheumatoid arthritis [43], a point which was confirmed by the results of Gheita et al. study in 2012 [44].

Considering the importance of phlebitis induced by intravenous injection of chemotherapy drugs and the consequence of imposing additional treatment costs for the patient and the health system, the present study was designed to determine the effect of N. *sativa* oil on the prevention of phlebitis induced by chemotherapy.

## 2. Materials and methods

### 2.1. Study design and registration

The present study was a single-blind (patients) clinical trial performed on 60 patients undergoing chemotherapy admitted to the Oncology Department of Tohid Hospital (affiliated to kurdistan University of Medical Sciences) in Sanandaj city, from July to December of 2018. This study was approved by Ethics Committee of Islamic Azad University, Tehran Medical Sciences Branch; the clinical trial protocol was registered at the Iranian Registry of Clinical Trials (IRCT) under No: IRCT20181208041892N1. The CONSORT checklist was used to report the study [45]. Written and oral informed consent obtained from all participants.

### 2.2. Eligibility criteria

Inclusion criteria were Being aged 25 to 75 years, full awareness and collaboration to participate in the research, having gastrointestinal cancers (colon, rectum, esophagus, stomach), being on chemotherapy diets such as 5-fluorouracil +oxaloplatin+ Leucovorin or 5-fluorouracil +Cisplatin, application of 20-22 catheter gauge (made of Unicut Company), use of upper limb for inserting of intravenous catheter, non-use of analgesics and pain relief, non-steroidal anti-inflammatory drugs, non-use of antibiotics, Lack of diabetes, skin disorders and autoimmune diseases, lack of fever, neutropenia and any signs of infection, not using any ointment or vegetable oil at the site of venipuncture during research were the main inclusion criteria. Exclusion criteria included the patient’s unwillingness to continue to collaborate in the research, early discharge, ill-being and allergic reactions during the study.

### 2.3. Intervention

**Fig. 1:**
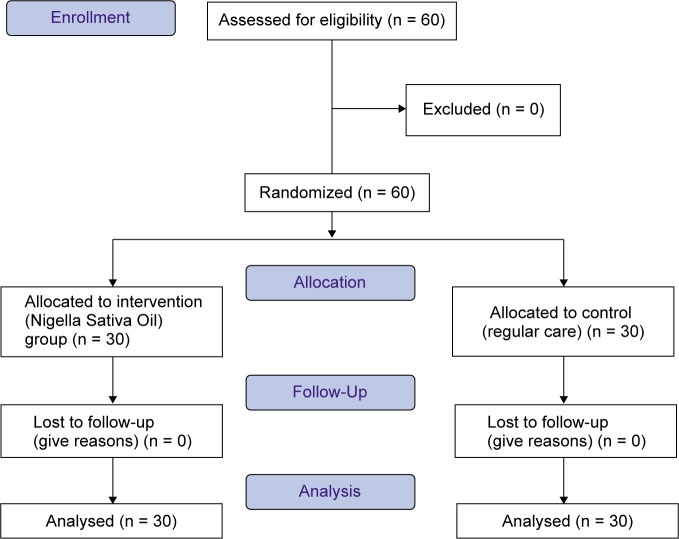
CONSORT 2010 flow diagram.

The sample size, derived from the same study [21] considering to 80% power of test and 5% probability of type 1 error, the sample size were calculated 30 subjects in each group. This study was single blind, patients were aware of being involved in the study, but not aware of which group they are in at the time of intervention. First the researcher providing explanations about the method and the objective of the research to the all patients. In this study, the samples were selected by Convenience Sampling method and then, were randomly assigned into intervention (N. *sativa* oil) group and control (routine care) groups. In intervention group, the participants (30 patients), initially, Angiocath IV Catheter, 20-22 gauge sizes (manufactured by UNICUT company) was inserted in the upper limb veins of the participants, observing the aseptic Technique. After the insertion of peripheral vein catheter, 5 drops of N. *sativa* (prepared by the pharmacist) was applied 2 times a day (each 12 hours, morning and night before bedtime) on the distal area of the catheter up to 10 cm on the route of intravenous injection from the first to the third day of chemotherapy cycle in the intervention group. Also, the place was washed with water each time before using oil; No intervention was conducted for the participants in the control group. The participants of two groups were evaluated for incidence and severity of phlebitis in these three days every 12 hours at intervals of 12, 24, 36, 48, 60, 72 hours. On the first day, all the participants were given the same training for catheter care. Collected data was analyzed by SPSS 21 using descriptive statistics (mean, standard deviation) and descriptive statistics (Chi-Square, Fisher exact, T-test, MannWhitney test). (Figure 1)

### 2.4. Data collection and instruments

Data were collected using a two-part questionnaire. The first part included demographic characteristics of patients, including age, sex, marital status, educational level, occupational status, place of residence, and BMI; the second part of the questionnaire contained information about the disease,intravenous therapy and catheter characteristics, including the duration of the disease, the duration of chemotherapy, chemotherapy cycles, chemotherapy diet (type, amount, duration and method of administration), absolute neutrophil count, use of steroid anti-inflammatory drugs, history of underlying diseases, catheter insertion date and time, catheter gauge and anatomical location of the catheter. In addition, the phlebitis checklists, based on Visual Infusion Phlebitis (VIP) [46], were used to assess the place of the catheter insertion in terms of the incidence and severity of phlebitis at 12, 24, 36, 48, 60, 72 hour intervals. In order to determine the scientific validity of the questionnaire, the content validity method was used and approved by 10 faculty members specializing in the field under study; the reliability between two evaluatros was used to assess the reliability of the phlebitis check list; and the reliability coefficient was estimated to be 0.85 by the Kappa test.

## 3. Results

60 subjects participated in the present study. 53.3% of the subjects in the intervention and 63.3% in the control group were male and the rest of the subjects were female. The mean age in the intervention group was 58.9 ± 12.29 and 62.93 ± 12.87 in the control group. The distribution of the subjects was similar in terms of demographic characteristics and disease characteristics such as age, sex, marital status, educational level, body mass index, type of cancer, duration of chemotherapy, chemotherapy cycles, and there was no significant difference between two groups (P > 0.05). There was no significant relationship between age, sex, catheter insertion, catheter gauge and type of chemotherapy diet with phlebitis in the present study. (Table 1)

**Table 1:** Comparison of demographic and disease characteristics in two groups of intervention and control.

variables		Group		*p* value
		control group n (%)	intervention group n (%)	
‘Age	25-37	2 (6.7)	2 (6.7)	**0.38**
	38-50	(3/3) 1	(16/7) 5	
	51-63	(36/7) 11	(33/3) 10	
	64-75	(53/3) 16	(43/3) 13	

Sex	male	(36/7) 11	(46/7) 14	**0.43**
	female	(63/3) 19	(53/3) 16	

BMI	>18/5	(33/3) 10	(16/7) 5	**0.25**
	18/5-25	(56/7) 17	(63/3) 19	
	25-30	(10) 3	(20) 6	

type of cancer	Colon	(53/3) 16	(50) 15	**0.999**
	Rectum	(3/3) 1	(6/7) 2	
	esophagus	(23/3)7	(23/3) 7	
	Stomach	(20) 6	(20) 6	

Type of chemotherapy diet	5-fluorouracil- oxaloplatin- leucovorin	(73/3) 22	(80) 24	**0.54**
	5-Fluorouracil- Cisplatin	(26/7) 8	(20) 6	

Catheter gauge	20	(30) 9	(46/7) 14	**0.18**
	22	(70) 21	(53/3) 16	

	Back of the hand	(10) 3	(10) 3	**0.34**

Catheter insertion site	Forearm	(70) 21	(50) 15	
	Wrist	(10) 3	(26/7) 8	
	Elbow	(10) 3	(13/3) 4	

*Chi-square* test did not show a significant difference between two groups in terms of phlebitis incidence at 12 hours (P = 0.2), 24 (P = 0.8), 36 (P = 0.4) and 48 hours (P = 0.06); however, this difference was statistically significant at 60 (P = 0.04) and 72 (P = 0.001) hours. (Table 2)

**Table2:** Frequency distribution of phlebitis incidence in the studied groups by time intervals.

incidence of phlebitis		yes		no		*p* value
		%	N	%	N	
12	Intervention	36/7	11	63/3	19	**0.19**
	Control	53/3	16	46/7	14	

24	Intervention	50	15	50	15	**0.79**
	Control	53/3	16	46/7	14	

36	Intervention	43/3	13	56/7	17	**0.43**
	Control	53/3	16	46/7	14	

48	Intervention	33/3	10	66/7	20	**0.06**
	Control	56/7	17	43/3	13	

60	Intervention	30	9	70	21	**0.037**
	Control	56/7	17	43/3	13	

72	Intervention	23/3	7	76/7	23	**0.001**
	Control	66/7	20	33/3	10	

Based on Fisher’s exact test, at 12 (P = 0.02) and 72 (P = 0.001) hours, there was a significant difference between the two groups in regard to the severity of phlebitis. There was no statistically significant difference between the two groups at 24 (P = 0.1), 36 (*P* = 0.1), 48 (P = 0.2) and 60 (P = 0.056) hours. (Table 3)

**Table 3:** Frequency distribution of phlebitis severity in the studied groups by time intervals.

	Severity of phlebitis					
	Zero-degree (%)	One-degree (%)	two-degree (%)	three-degree (%)	four-degree (%)	*p* value
Time (hour)						
12	Intervention	19	8	3	0	0	**0.02**
		(63/3)	(26/7)	(10)	(0)	(0)	

	Control	14	4	6	6	0	
		(46/7)	(13/3)	(20)	(20)	(0)

24	Intervention	15	8	5	2	0	**0.10**
		(50)	(26/7)	(16/7)	(6/7)	(0)	

	Control	14	13	0	3	0	
		(46/7)	(43/3)	(0)	(10)	(0)

36	Intervention	17	8	5	0	0	**0.16**
		(56/7)	(26/7)	(16/7)	(0)	(0)	

	Control	14	4	11	1	0	
		(46/7)	(13/3)	(36/7)	(3/3)	(0)

48	Intervention	20	7	3	0	0	**0.19**
		(66/7)	(23/3)	(10)	(0)	(0)	

	Control	13	9	6	2	0	
		(43/3)	(30)	(20)	(6/7)	(0)

60	Intervention	21	6	3	0	0	**0.056**
		(70)	(20)	(10)	(0)	(0)	

	Control	13	6	10	1	0	
		(43/3)	(20)	(33/3)	(3/3)	(0)

72	Intervention	23	5	2	0	0	**0.001**
		(76/7)	(16/7)	(6/7)	(0)	(0)	

	Control	10	5	11	3	1	
		(33/3)	(16/7)	(36/7)	(10)	(3/3)

Also, the Mann-Whitney test showed a significant difference in mean phlebitis severity at 12, 48, 60 and 72 hours; however, this difference was not significant at 24 and 36 hours. (Table 4)

**Table 4:** Mean and standard deviation of phlebitis severity in the studied groups by time intervals.

	The mean severity of phlebitis				
	intervention		control	
Time (hour)	mean (severity of phlebitis)	SD	mean (severity of phlebitis)	SD	*p* value
12	0/47	0/68	1/13	1/22	0/04
24	0/80	0/96	0/73	0/91	0/859
36	0/60	0/77	0/96	0/99	0/167
48	0/43	0/67	0/90	0/95	0/044
60	0/40	0/67	0/97	0/96	0/016
72	0/30	0/59	1/33	1/15	0/0001

## 4. Discussion

The results of the study showed that the difference between the two groups turned out to be significant in terms of the phlebitis incidence from 60 hours onwards, and the most significant difference was observed at 72 hours, a time which the incidence of phlebitis was observed on its maximum value in the control group; 23 subjects in the intervention group and 10 subjects in the control group did not develop phlebitis at this time of study. During the 72 hours of study, the severity of phlebitis reached the lowest possible rate in the intervention group, but the severity of phlebitis increased in the control group, so that a fourth degree phlebitis was observed. The results of the findings indicated the positive effect of nigella sativa oil on the incidence and severity of phlebitis in the intervention group over time, as well as increased life span of the peripheral vein catheter in this group compared to the control group. In this regard, Mosayebi *et al.* (2017), in a study conducted to determine the effect of sesame oil on the prevention of chemotherapy-induced phlebitis in children with acute lymphoblastic leukemia, found that there was a significant difference between the two groups in terms of phlebitis incidence and mean phlebitis severity [21]. The results of this study indicated that sesame oil was effective in preventing phlebitis caused by chemotherapy, a point which was, also, confirmed by the results of Nekozad study [47]. Also the results of a study by Thakur showed that after 3 days of chemotherapy cycle, 85% of the subjects in the control group and 40% in the test group developed thrombophlebitis. The findings of the study showed a significant difference in chemotherapy induced thrombophlebitis between the two groups. These findings are consistent with results of the present study [24]. Also The results of a study by Hamabe et al. showed a reduction of phlebitis in patients receiving steroid ointment with Vaseline, and found that the combination of steroid ointment with Vaseline ointment is effective in reducing the incidence and prevention of phlebitis caused by chemotherapy [23]. The results of a study by Ravindra et al. showed that after intervention, the mean phlebitis severity was 1.10 in the intervention group and 2.53 in the control group, which showed a statistically significant difference between the two groups in terms of the level and severity of phlebitis [18]. Jing Hong found that fresh potato cuttings was more effective than hirodoid ointment in the treatment of amiodarone phlebitis [48]. In a review study, Gao *et al.* concluded that topical aloe vera was more effective than compared to magnesium sulfate glycerin in preventing and treating chemotherapy-induced phlebitis [1]. Several studies have been done on the therapeutic and anti-inflammatory properties of nigella N. *sativa,* Kooshki study realized that topical application of N. *sativa* oil was more effective in reducing pain in the elderly’s knee compared to acetaminophen tablets; thus, it is suggested as an alternative remedy for elderly people [49]. The results of a study by Yousefy showed that N. *sativa* had similar effects with betamethasone ointment in reducing the severity of hand eczema and improving skin quality [35].

In a study conducted by Ghorbani Birgani,after 6 months of intervention, the mean vitiligo location scores decreased from 4.98 ± 4.81 to 3.75 ± 3.91 in the N. *sativa* seed group; this rate decreased from 4.98 ± 4.80 to 4.62 ± 4.36 in the group receiving fish oil, indicating that N. *sativa* was more effective in improving skin lesions compared to fish oil after 6 months of intervention [33].

## 5. Conclusion

According to the results of the current study, topical application of N. *sativa* oil is effective in the prevention of chemotherapy-induced phlebitis as a simple and accessible method without known side effects, it is recommended in prevention of phlebitis induced by chemotherapy. Additionally, further research is recommended with larger sample size and in other wards of the hospital.

## Conflicts of interest statement

None.

## References

[1] Gao Y , Jiang T , Mei S , Zhang S , Zhu C , Sun Y . Meta-analysis of Aloe vera for the prevention and treatment of chemotherapy-induced phlebitis. Int J Clin Exp Med. 2016; 9(6): 9642–50.

[2] Gilbert RE , Kozak MC , Dobish RB , Bourrier VC , Koke PM , Kukreti V , et al Intravenous Chemotherapy Compounding Errors in a Follow-Up Pan-Canadian Observational Study. J Oncol Pract. 2018; 14(5): e295–e303. 29676947 10.1200/JOP.17.00007 PMC5952328

[3] Kapucu S , Ozkaraman AO , Uysal N , Bagcivan G , Çeref FÇ , Elôz A . Knowledge level on administration of chemotherapy through peripheral and central venous catheter among oncology nurses. Asia Pac J Oncol Nurs. 2017; 4(1): 61–8. 28217732 10.4103/2347-5625.199081 PMC5297235

[4] Vinograd AM , Zorc JJ , Dean AJ , Abbadessa MKF , Chen AE . First-attempt success, longevity, and complication rates of ultrasound-guided peripheral intravenous catheters in children. Pediatr Emerg Care. 2018; 34(6): 376–80. 28221281 10.1097/PEC.0000000000001063

[5] Marsh N , Webster J , Larson E , Cooke M , Mihala G , Rickard CM . Observational Study of Peripheral Intravenous Catheter Outcomes in Adult Hospitalized Patients: A Multivariable Analysis of Peripheral Intravenous Catheter Failure. J Hosp Med. 2018; 13(2): 83–9. 29073316 10.12788/jhm.2867

[6] Pasalioglu KB , Kaya H . Catheter indwell time and phlebitis development during peripheral intravenous catheter administration. Pak J Med Sci. 2014; 30(4): 725–30. 25097505 PMC4121686

[7] Chau CT , Prielipp RC , Wahr JA . Prevention of Thrombophlebitis in Peripheral Intravenous Catheters: The Butterfly Effect. LWW, 2018. 10.1213/ANE.0000000000003764 30433918

[8] Alexandrou E , Ray-Barruel G , Carr PJ , Frost S , Inwood S , Higgins N , et al Use of short peripheral intravenous catheters: characteristics, management, and outcomes worldwide. J Hosp Med. 2018; 13(5). 29813140 10.12788/jhm.3039

[9] Urbanetto JdS , Peixoto CG , May TA . Incidence of phlebitis associated with the use of peripheral IV catheter and following catheter removal. Rev Lat Am Enfermagem. 2016; 24. 10.1590/1518-8345.0604.2746 PMC4990043 27508916

[10] Guihard B , Rouyer F , Serrano D , Sudrial J , Combes X . Appropriateness and Complications of Peripheral Venous Catheters Placed in an Emergency Department. J Emerg Med. 2018; 54(3): 281–6. 29217204 10.1016/j.jemermed.2017.10.005

[11] Liu L , Su SW , Zhou P , Song R , Sun HY . External application of moisture exposed burn ointment for phlebitis: A meta-analysis of randomized controlled trials. Int J Med Med Sci. 2017; 9(12): 158–73.

[12] Yoshida Y , Hoshino S , Aisu N , Naito M , Tanimura S , Mogi A , Tanaka T , Hirata K , Tamura K , Yamashita Y . Administration of chemotherapy *via* the median cubital vein without implantable central venous access ports: port-free chemotherapy for metastatic colorectal cancer patients. Int J Clin Oncol. 2015; 20(2): 332–7. 24811333 10.1007/s10147-014-0703-5

[13] Damanik C . The Effectiveness of Sesame Oil against Pain Intensity of Phlebitis in Cancer Patients undergoing Chemotherapy. Philippine J Med, 1(4).

[14] Bertoglio S , van Boxtel T , Goossens GA , Dougherty L , Furtwangler R , Lennan E , Pittiruti M , Sjovall K , Stas M . Improving outcomes of short peripheral vascular access in oncology and chemotherapy administration, SAGE Publications Sage UK: London, England, 2017: 2019. 10.5301/jva.5000668 28127726

[15] Tzolos E , Salawu A . Improving the frequency of visual infusion phlebitis (VIP) scoring on an oncology ward. BMJ Open Quality. 2014; 3(1): u205455 w2364. 10.1136/bmjquality.u205455.w2364 PMC4645857 26734282

[16] Beccaria LM , Machado BD , dos santos Bertolli E , Contrin LM , Werneck AL . Incidence of phlebitis in adult patients. J Nurs UFPE on line. 2018; 12(3): 745–52.

[17] Reis PEDd , Carvalho ECd , Bueno PCP , Bastos JK . Clinical application of Chamomilla recutita in phlebitis: dose response curve study. Rev Lat Am Enfermagem. 2011; 19(1): 3–10. 21412623 10.1590/s0104-11692011000100002

[18] Ravindra H . A quasi experimental study to evaluate effectiveness of glycerin magnesium sulphate dressing on phlebitis among patients undergoing peripheral intravenous infusion in selected hospital, Vadodara. Int J Med Res Health Sci. 2015; 4(3): 527–30.

[19] Sheikhi A , Asadizaker M , Jahani S , Koochak M , Shamloo MBB , Zadeh MHH . The effect of rosemary topical ointment on phlebitis caused by antibiotic therapy in intensive care units. J Int J Pharm Re. 2018; 45: 5–11.

[20] Shen J , Zhang J , Yin W , Ligao W . Application Status of Phytotherapy for Prevention and Treatment of Chemotherapy Induced Phlebitis. Chin J Infor Trad Chin Med. 2016; 23(6): 134–6.

[21] Mosayebi N , Sharifpour Z , Asgari F , Atrkarroushan Z , Pasdaran A . The Efficacy and Safety of Sesame Oil in Prevention of Chemotherapy-Induced Phlebitis in Children with Acute Lymphoblastic Leukemia. Iran J Ped Hematol Oncol. 2017; 7(4): 198–206.

[22] Zheng GH , Yang L , Chen HY , Chu JF , Mei L . Aloe vera for prevention and treatment of infusion phlebitis. Cochrane Database Syst Rev. 2014; 6. 10.1002/14651858.CD009162.pub2 PMC6464352 24895299

[23] Hamabe Y , Hanai A , Ishiguro H , Kuroda T , Hirota M , Nomura M , Ishikawa H , Muto M . 5040 Effects of steroid ointment application on chemotherapy-induced phlebitis: A randomized, double-blind, placebo-controlled clinical trial. Ann Oncol. 2017; 28(10): mdx676. 003.

[24] Thakur R . Effectiveness of Heparin lock flush on Chemotherapy Induced Thrombophlebitis among Patients receiving Chemotherapy. Res J Recent Sci. 2015; 4: 177–80.

[25] Panahpour H , Golmohammadi M , Mohamadnejad S . Effects of the Treatment with Nigella sativa Oil on Brain Injury and Edema in Experimental Model of Stroke in Rats. J Ardabil Univ Med Sci. 2015; 15(3): 301–10.

[26] Chaudhry H , Fatima N , Ahmad IZ . Evaluation of antioxidant and antibacterial potentials of Nigella sativa L. suspension cultures under elicitation. Biomed Res Int. . 2015: 2015. 10.1155/2015/708691 PMC4549534 26347883

[27] Agbaria R , Gabarin A , Dahan A , Ben-Shabat S . Anticancer activity of Nigella sativa (black seed) and its relationship with the thermal processing and quinone composition of the seed. Drug design, development and therapy. 2015; 9: 3119. 10.2147/DDDT.S82938 PMC4476428 26124636

[28] Al-Ghamdi M . The anti-inflammatory, analgesic and antipyretic activity of Nigella sativa. J Ethnopharmacol. 2001; 76(1): 45–8. 11378280 10.1016/s0378-8741(01)00216-1

[29] Dwarampudi LP , Palaniswamy D , Nithyanantham M , Raghu P . Antipsoriatic activity and cytotoxicity of ethanolic extract of Nigella sativa seeds. Pharmacognosy magazine. 2012; 8(32): 268. 24082629 10.4103/0973-1296.103650 PMC3785163

[30] Al-Harchan NA-AH . Treatment of Acne Vulgaris with Nigella sativa oil lotion. Iraqi Acad Sci J. 2010; 9(2): 140–4.

[31] Houghton PJ , Zarka R , de las Heras B , Hoult J . Fixed oil of Nigella sativa and derived thymoquinone inhibit eicosanoid generation in leukocytes and membrane lipid peroxidation. Planta medica. 1995; 61(1): 33–6. 7700988 10.1055/s-2006-957994

[32] Ali SA , Meitei KV . Nigella sativa seed extract and its bioactive compound thymoquinone: the new melanogens causing hyperpigmentation in the wall lizard melanophores. J Pharm Pharmacol. 2011; 63(5): 741–6. 21492177 10.1111/j.2042-7158.2011.01271.x

[33] Ghorbanibirgani A , Khalili A , Rokhafrooz D . Comparing Nigella sativa oil and fish oil in treatment of vitiligo. Iran Red Crescent Med J. 2014; 16(6). 10.5812/ircmj.4515 PMC4102993 25068060

[34] Chakravarty N . Inhibition of histamine release from mast cells by nigellone. Annals of allergy. 1993;70(3): 237–42. 7680846

[35] Yousefi M , Barikbin B , Kamalinejad M , Abolhasani E , Ebadi A , Younespour S , et al Comparison of therapeutic effect of topical Nigella with Betamethasone and Eucerin in hand eczema. J Eur Acad Dermatol Venereol. 2013; 27(12): 1498–504. 23198836 10.1111/jdv.12033

[36] El-Kadi A , Kandil O . Effect of Nigella sativa (the black seed) on immunity, Proceeding of the 4th International Conference on Islamic Medicine, Kuwait. Bull Islamic Med 1986, 344–8.

[37] Mabrouk G , Moselhy S , Zohny S , Ali E , Helal T , Amin A , et al Inhibition of methylnitrosourea (MNU) induced oxidative stress and carcinogenesis by orally administered bee honey and Nigella grains in Sprague Dawely rats. J Exp Clin Cancer Res. 2002; 21(3): 341–6. 12385575

[38] Zafar K , Noorul H , Nesar A , Vartika S , Khalid M , Prashant S , et al Pharmacological activity of Nigella sativa: A review. World J. Pharm. Sci. 2016; 45: 234–41.

[39] Khazdair MR . The protective effects of Nigella sativa and its constituents on induced neurotoxicity. J Toxicol. 2015; 2015. 10.1155/2015/841823 PMC4641935 26604923

[40] Randhawa MA , Alenazi SA . Neuropsychiatric Effects of Nigella sativa (Black Seed) - A Review. Altern Integr Med. 2016; 5(1): 1–8.

[41] Burits M , Bucar F . Antioxidant activity of Nigella sativa essential oil. Phytotherapy research. 2000; 14(5): 323–8. 10925395 10.1002/1099-1573(200008)14:5<323::aid-ptr621>3.0.co;2-q

[42] Nergiz C , Otle§ S. . Chemical composition of Nigella sativa L. seeds. Food chemistry. 1993; 48(3): 259–61.

[43] Hadi V , Kheirouri S , Alizadeh M , Khabbazi A , Hosseini H . Effects of Nigella sativa oil extract on inflammatory cytokine response and oxidative stress status in patients with rheumatoid arthritis: a randomized, double-blind, placebo-controlled clinical trial. Avicenna J Phytomed. 2016; 6(1): 34. 27247920 PMC4884216

[44] Gheita TA , Kenawy SA . Effectiveness of Nigella sativa oil in the management of rheumatoid arthritis patients: a placebo controlled study. Phytother Res. 2012; 26(8): 1246–8. 22162258 10.1002/ptr.3679

[45] Schulz KF , Altman DG , Moher D . CONSORT 2010 statement: updated guidelines for reporting parallel group randomised trials. BMC medicine. 2010; 8(1): 18. 20334633 10.1186/1741-7015-8-18 PMC2860339

[46] Gallant P , Schultz AA . Evaluation of a visual infusion phlebitis scale for determining appropriate discontinuation of peripheral intravenous catheters. J Infus Nurs. 2006; 29(6): 338–45. 17122689 10.1097/00129804-200611000-00004

[47] Nekozad N , Ashktorab T , Mojab F , Alavi H , Azadeh P . The preventative role of sesame oil on phlebitis induced by anti-neoplastic agents. EBNESINA-Journal of Medical. 2011; 14(1, 2): 10–6.

[48] J.-H. L.. Comparison between two methods of preventing Amiodaro- neinduced phlebitis. S China. J Cardiology. 2014; 15(3): 198–202.

[49] Kooshki A , Forouzan R , Rakhshani MH , Mohammadi M . Effect of topical application of Nigella sativa oil and oral acetaminophen on pain in elderly with knee osteoarthritis: a crossover clinical trial. Electron Physician. 2016; 8(11): 3193. 28344755 10.19082/3193 PMC5358924

