# Correction notice to: The effect of nigella *sativa* oil on the prevention of phlebitis induced by chemotherapy: a clinical trial

**DOI:** 10.1051/bmdcn/2019090430

**Published:** 2019-11-14

**Authors:** Niaz Behnamfar, Zohreh Parsa Yekta, Faraz Mojab, Seyyed Mohammad Kazem Naeini

**Affiliations:** 1 Department of Nursing, Faculty of Nursing and Midwifery, Tehran Medical Sciences, Islamic Azad University Tehran Iran; 2 Department of Nursing management, Faculty of Nursing and Midwifery, Tehran Medical Sciences, Islamic Azad University Tehran Iran; 3 Department of Pharmacognosy, School of Pharmacy, Shahid Beheshti University of Medical Sciences Tehran Iran; 4 Department of Biostatistics, Tehran Medical Sciences, Islamic Azad University Tehran Iran

The authors have informed the journal about several errors in the published version of this article, which they missed to correct before publication. These errors occur in the article in the following parts: Table 1 and Table 3.

Futher, on page 33, in 2.3. Intervention, considering the 80% probability of testing, and the type 1 error of 5% is changed to considering the 80% power of test and 5% probability of type 1 error. In addition, on page 34, 58.9 ± 2.24 and 62.93 ± 2.35 should be 58.9 ± 12.29 and 62.93 ± 12.87.

Finally, the affiliation is changed from Department of Nursing and midwifery to Department of Nursing. In the new version all these tables are corrected. The authors, editors and the publisher apologize for these inconveniences.

